# Analysis of Biological Aging and Risks of All-Cause and Cardiovascular Disease–Specific Death in Cancer Survivors

**DOI:** 10.1001/jamanetworkopen.2022.18183

**Published:** 2022-06-22

**Authors:** Dongyu Zhang, Christiaan Leeuwenburgh, Daohong Zhou, Yan Gong, Marco Pahor, Jonathan D. Licht, Dejana Braithwaite

**Affiliations:** 1 Department of Epidemiology, University of Florida College of Public Health and Health Professions, Gainesville; 2 University of Florida Health Cancer Center, Gainesville; 3 Department of Aging and Geriatric Research, University of Florida College of Medicine, Gainesville; 4 Department of Biochemistry and Structural Biology, University of Texas Health Science Center at San Antonio; 5 Department of Pharmacotherapy and Translational Research, University of Florida College of Pharmacy, Gainesville; 6 Division of Hematology and Oncology, University of Florida Health Cancer Center, Gainesville; 7 Department of Surgery, University of Florida College of Medicine, Gainesville

## Abstract

This cohort study examines the association between biological aging and all-cause and cardiovascular disease–specific death rates among individuals with cancer.

## Introduction

Cancer is an age-related disease. In the United States, the median age at cancer diagnosis is 66 years.^1^ In older people diagnosed with cancer, aging^2^ and cancer treatment toxic effects^3^ are associated with unfavorable prognoses, such as death and adverse cardiovascular outcomes, which are the cause of death for more than 10% of individuals who had cancer.^4^ Here, we used National Health and Nutrition Examination Survey (NHANES) data to investigate the association between biological aging (BA) and risk of all-cause and cardiovascular disease (CVD)–specific mortality in cancer survivors; we hypothesized that cancer survivors with high BA would have a higher risk of all-cause and CVD-specific mortality.

## Methods

The University of Florida determined that this cohort study did not require institutional review board approval because it used a publicly deidentified data set. NHANES participants provided written informed consent. The study followed the STROBE reporting guideline.

We used the 1999 to 2010 NHANES. The study population consisted of adults with cancer history surviving for 1 year or more after diagnosis and an age-matched cohort without cancer history. We first estimated Levine phenotypic age and then the residual from a linear regression, which was treated as BA (eMethods and eTable in the Supplement); prior research showed that Levine phenotypic age was associated with mortality even after adjusting for chronological age, and BA value represented acceleration of Levine phenotypic age.^5^ BA was categorized as an ordinal variable (less than −5.07, −5.07 to less than −1.26, −1.26 to less than 3.94, and 3.94 or greater) to approximate population quartiles, with a larger positive value suggesting higher BA. We summarized BA in cancer survivors, the matched cohort, and subpopulations defined by sex, self-reported race and ethnicity, and cancer type using mean and 95% CI. Participants were censored if they were alive for all-cause mortality and if they died from non-CVD causes for CVD-specific mortality. Trend tests were conducted treating BA as a continuous variable. Participants without missing values for BA, death, or other covariates were included.

## Results

Among 2002 cancer survivors (53.8% women; 12.1% non-Hispanic Black, 75.5% non-Hispanic White, and 12.4% other race or ethnicity) and 2002 participants in the matched cohort (50.8% women; 16.9% non-Hispanic Black, 55.8% non-Hispanic White, and 27.3% other race or ethnicity), the mean (SD) age was 64.7 (14.9) years. Individuals with cancer history were older, and Black survivors were biologically older than White survivors (Figure). The median follow-up was 8.6 and 8.2 years for survivors and the matched cohort, respectively. The percent increase in mortality rate (highest vs lowest quartile of BA) was higher in survivors than the matched cohort (all cause: 189% vs 119%; CVD: 157% vs 109%). In log-rank tests, all-cause and CVD-specific mortality increased with BA in both groups (Table). In cancer survivors, BA (≥3.94 vs −5.07) was associated with increased risk of all-cause (adjusted HR [aHR], 2.65; 95% CI, 2.09-3.35; *P* for trend < .001) and CVD-specific mortality (aHR, 2.30; 95% CI, 1.37-3.85; *P* for trend = .01). The association pattern was similar for the matched cohort, although HRs were lower (all cause: aHR, 2.37; 95% CI, 1.86-3.03; *P* for trend < .001; CVD: aHR, 2.11; 95% CI, 1.27-3.49; *P* for trend = .007). Correction for sampling weight did not substantially change the results (Table).

**Figure.**
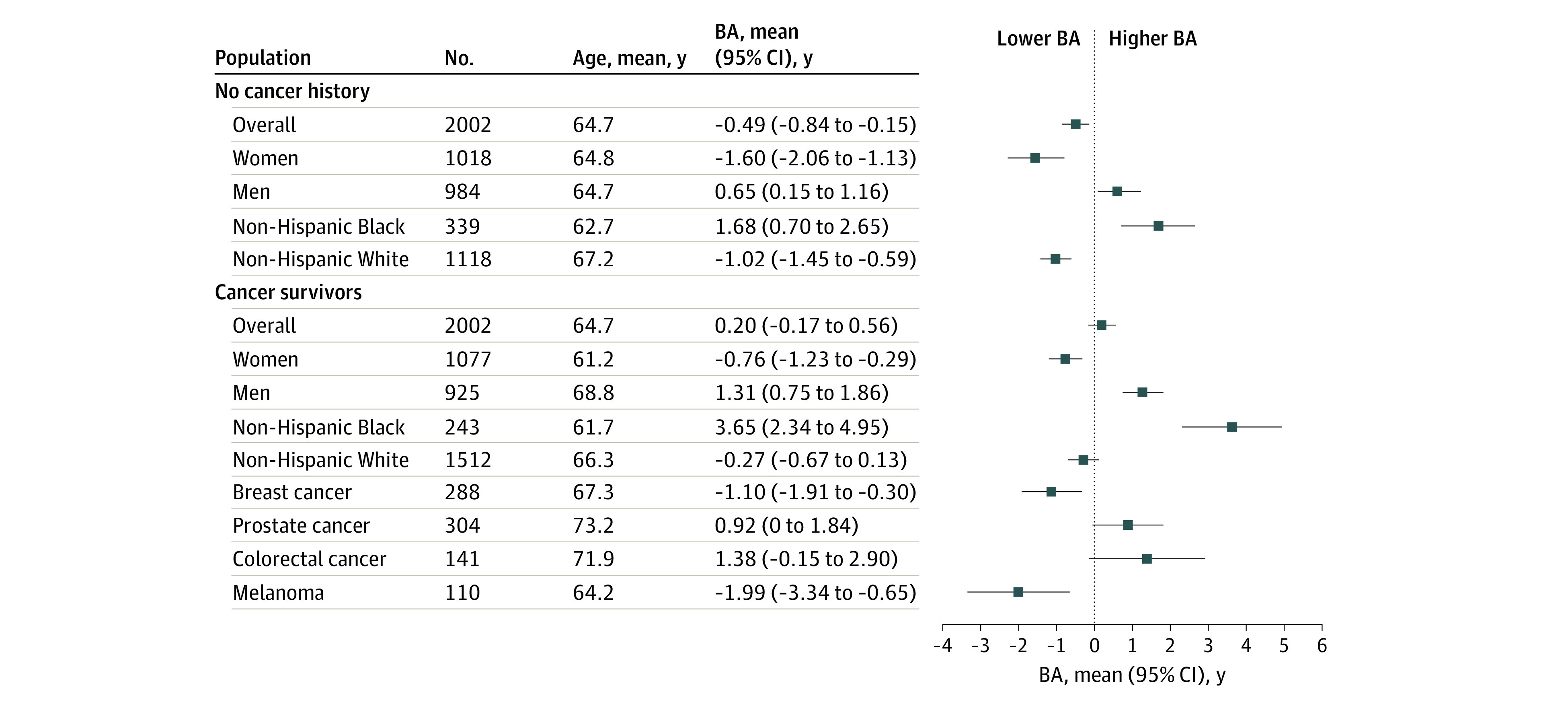
Forest Plot of Biological Aging (BA) by Subpopulation The figure shows number of participants, mean age at baseline, and mean value with 95% CI of BA by subpopulation. For sample size consideration, BA was summarized only for non-Hispanic White and Black participants when summaries were conducted by racial and ethnic group.

**Table.** Association Between BA and Mortality

BA, y	Adults, No. (N = 4004)	Deaths, No./PY	Mortality rate, No./1000 PY (95% CI)	Log-rank *P*	Unadjusted model, HR (95% CI)	*P* for trend	Adjusted model 1, aHR (95% CI)^a^	*P* for trend	Adjusted model 2, aHR (95% CI)^b^	*P* for trend
**All-cause mortality**										
With cancer history										
<−5.07	501	119/4844.1	24.6 (20.5-29.4)	<.001	1 [Reference]	<.001	1 [Reference]	<.001	1 [Reference]	<.001
−5.07 to <−1.26	499	120/4584.9	26.2 (21.9-31.3)		1.08 (0.84-1.40)		1.11 (0.86-1.43)		1.16 (0.87-1.56)	
−1.26 to <3.94	503	170/4208.8	40.4 (34.8-46.9)		1.73 (1.37-2.19)		1.58 (1.24-2.00)		1.67 (1.23-2.26)	
≥3.94	499	245/3450.6	71.0 (62.6-80.5)		3.20 (2.56-3.99)		2.65 (2.09-3.35)		3.04 (2.31-4.00)	
Overall	2002	654/17 088.3	38.3 (35.4-41.3)	NA	NA	NA	NA	NA	NA	NA
Without cancer history										
<−5.07	575	132/5611.9	23.5 (19.9-27.9)	<.001	1 [Reference]	<.001	1 [Reference]	<.001	1 [Reference]	<.001
−5.07 to <−1.26	516	118/4888.8	24.1 (20.2-28.9)		1.04 (0.81-1.33)		1.10 (0.86-1.42)		1.44 (1.06-1.94)	
−1.26 to <3.94	480	125/4343.8	28.8 (24.1-34.3)		1.26 (0.99-1.61)		1.24 (0.96-1.60)		1.51 (1.10-2.06)	
≥3.94	431	175/3405.3	51.4 (44.3-59.6)		2.37 (1.89-2.98)		2.37 (1.86-3.03)		2.68 (1.89-3.80)	
Overall	2002	550/18 249.8	30.1 (27.7-32.8)	NA	NA	NA	NA	NA	NA	NA
**CVD-specific mortality**										
With cancer history										
<−5.07	501	26/4844.1	5.4 (3.7-7.9)	<.001	1 [Reference]	<.001	1 [Reference]	.001	1 [Reference]	.02
−5.07 to <−1.26	499	22/4584.9	4.8 (3.2-7.3)		0.90 (0.51-1.60)		0.96 (0.54-1.70)		1.06 (0.54-2.07)	
−1.26 to <3.94	503	30/4208.8	7.1 (5.0-10.2)		1.39 (0.82-2.35)		1.27 (0.74-2.17)		1.36 (0.71-2.61)	
≥3.94	499	48/3450.6	13.9 (10.5-18.5)		2.85 (1.76-4.62)		2.30 (1.37-3.85)		2.42 (1.21-4.82)	
Overall	2002	126/17 088.3	7.4 (6.2-8.8)	NA	NA	NA	NA	NA	NA	NA
Without cancer history										
<−5.07	575	31/5611.9	5.5 (3.9-7.9)	.01	1 [Reference]	.002	1 [Reference]	.007	1 [Reference]	.01
−5.07 to <−1.26	516	36/4888.8	7.4 (5.3-10.2)		1.34 (0.83-2.16)		1.40 (0.86-2.28)		2.53 (1.43-4.49)	
−1.26 to <3.94	480	33/4343.8	7.6 (5.4-10.7)		1.39 (0.85-2.28)		1.35 (0.81-2.23)		1.87 (1.02-3.41)	
≥3.94	431	39/3405.3	11.5 (8.4-15.7)		2.17 (1.35-3.48)		2.11 (1.27-3.49)		2.92 (1.48-5.76)	
Overall	2002	139/18 249.8	7.6 (6.4-9.0)	NA	NA	NA	NA	NA	NA	NA

Abbreviations: aHR, adjusted hazard ratio; BA, biological aging; CVD, cardiovascular disease; HR, hazard ratio; NA, not applicable; PY, person-year.

^a^ The model was adjusted for sex, race and ethnicity, education, marital status, smoking status, body mass index, energy intake, burden of comorbidities (chronic kidney disease, osteoporosis, diabetes, arthritis, heart attack, coronary heart disease, stroke, hypertension, emphysema, chronic bronchitis, and congestive heart failure), and year of survey. For cancer survivors, the model was additionally adjusted for history of more than 1 cancer and time since cancer diagnosis.

^b^ The model was adjusted for the same set of covariates and corrected for the National Health and Nutrition Examination Survey sampling weight.

## Discussion

This cohort study found that, compared with individuals without a cancer history, individuals with a prior cancer diagnosis had increased BA, which was associated with increased all-cause and CVD-specific mortality. In multivariable models, point estimates in the association between BA and death were lower in individuals without cancer history, although their overlapping 95% CIs did not support statistically significant differences. Limitations of our study include the lack of cancer-specific variables, treatment information, and validation of self-reported cancer history. Biomarkers incorporated in BA were measures that could be obtained in routine clinical practice, suggesting that clinicians and health researchers may be able to leverage these real-world resources (eg, blood testing results in electronic health records) to monitor BA for cancer survivors, estimate risk of adverse events, and inform cancer survivors of prognosis more precisely.^6^ Future prospective studies should explore the association of interventions ameliorating BA in cancer survivors with decreased adverse outcomes in survivorship.

## Notes

Supplement.
eMethods.
**eTable.** Algorithm Used to Compute Biological Aging
eReferences.
