# Supplementary material for: Analysis of Biological Aging and Risks of All-Cause and Cardiovascular Disease–Specific Death in Cancer Survivors
Source: JAMA Netw Open. 2022 Jun 22;5(6):e2218183. doi: 10.1001/jamanetworkopen.2022.18183 (PMC9218849; doi:10.1001/jamanetworkopen.2022.18183)
Supplement: Supplement. — eMethods. eTable. Algorithm Used to Compute Biological Aging eReferences. [file jamanetwopen-e2218183-s001.pdf]

Supplemental Online Content

Zhang D, Leeuwenburgh C, Zhou D, et al. Analysis of biological aging and risks of all-cause and cardiovascular disease–specific death in cancer survivors. *JAMA Netw Open*. 2022;5(6):e2218183. doi:10.1001/jamanetworkopen.2022.18183

eMethods.

eTable. Algorithm Used to Compute Biological Aging

eReferences.

This supplemental material has been provided by the authors to give readers additional information about their work.

© 2022 Zhang D et al. *JAMA Network Open.*

eMethods

We used the 1999 to 2010 NHANES led by the Centers for Disease Control and Prevention. The NHANES uses interviews, physical examinations, and laboratory testing to measure participants' demographic and health-related factors.

To obtain BA, we first estimated Levine phenotypic age based on 9 blood biomarkers and chronological age. We then estimated the residual from a linear model regressing Levine phenotypic age on chronological age, and the value of the residual was treated as the measure of BA representing acceleration of phenotypic age.

Death was identified by linkage to the National Death Index through December 31, 2015, and *International Statistical Classification of Diseases and Related Health Problems, Tenth Revision* (*ICD-10*) was used to ascertain cause of death.

Log-rank test assessed if mortality rate differed by levels of BA. Unadjusted and multivariable Cox proportional hazards models estimated hazard ratio (HR) and 95% CI of all-cause and cardiovascular disease (CVD)–specific death for cancer survivors and matched cohort by BA category. Cox models were performed for cancer survivors and matched cohort separately; the proportionality assumption was examined by scaled Schoenfeld residuals, and there was no violation.

The multivariable model adjusted for sex, race and ethnicity, education, marital status, smoking status, body mass index, energy intake, burden of comorbidities, year of survey, history of more than 1 cancer (for survivors), and time since cancer diagnosis (for survivors). In a sensitivity analysis, the model was corrected for NHANES sampling weight and compared with the primary analysis.

In NHANES, race and ethnicity included non-Hispanic White, non-Hispanic Black, Mexican American, other Hispanic, and other race (including multiracial). For sample size consideration, we treated race and ethnicity as a 3-level variable in the multivariable Cox model, as non-Hispanic White, non-Hispanic Black, and other race or ethnicity. We incorporated race and ethnicity because age-related comorbidity burden varies by racial and ethnic group, suggesting that characteristics of aging may be heterogeneous by race and ethnicity.

In a sensitivity analysis, the model was corrected for the NHANES sampling weight and compared to the primary analysis.

© 2022 Zhang D et al. *JAMA Network Open.*

Statistical analyses were conducted in December 2021 with statistical software SAS version 9.4 (SAS Institute) and Stata version 16.0 (StataCorp).

© 2022 Zhang D et al. *JAMA Network Open.*

eTable. Algorithm Used to Compute Biological Aging

**Step 1**: Estimate phenotypic age using 9 biomarkers and chronological age<br><br>$$Phenotypic\ age = 141.50 + \frac{\ln\left[-0.00553 \times \ln(1-M)\right]}{0.09165}$$<br><br>$$\mathrm{M} = 1 - \exp\left(\frac{-1.51714 \times \exp\ (xb)}{0.0076927}\right)$$<br><br>xb=-19.907-0.0336×albumin (g/L) +0.0095×creatinine (µmol/L)+0.1953×glucose (mmol/L)+0.0954×ln (CRP) (g/L)-0.012×lymphocyte percent (%)+0.0268×mean cell volume (fL)+0.3306×red cell distribution width (%)+0.00188×alkaline phosphatase (U/L)+0.0554×white blood cell count (1000cell/µL)+0.0804×chronological age (year)
**Step 2**: Build a linear model regressing phenotypic age on chronological age
**Step 3**: Estimate residual from the linear model established in Step 2.<br><br>The residual value represents biological aging. It reflects whether a person appears older [positive value] or younger [negative value] than expected based on his/her age from a biological perspective.

© 2022 Zhang D et al. *JAMA Network Open.*

eReferences

Liu, Zuyun, et al. "Correction: a new aging measure captures morbidity and mortality risk across diverse subpopulations from NHANES IV: a cohort study." *PLoS medicine* 16.2 (2019): e1002760.

Liu, Zuyun, et al. "A new aging measure captures morbidity and mortality risk across diverse subpopulations from NHANES IV: a cohort study." *PLoS medicine* 15.12 (2018): e1002718.

© 2022 Zhang D et al. *JAMA Network Open.*
